# Application of a New Device for Vision Relaxation in Computer Users

**DOI:** 10.3390/vision8030040

**Published:** 2024-06-23

**Authors:** Aiga Svede, Svetlana Semjonova, Angelina Ganebnaya, Liga Puhova, Kulsum Fatima Baig, Alina Kucika, Gatis Ikaunieks, Karola Panke, Dmitry Gromov

**Affiliations:** 1 Department of Optometry and Vision Science, Faculty of Physics, Mathematics and Optometry, University of Latvia, Jelgavas Street 1, LV-1004 Riga, Latviaangelina.ganebnaya@lu.lv (A.G.); alina.kucika@lu.lv (A.K.); gatis.ikaunieks@lu.lv (G.I.); karola.panke@lu.lv (K.P.); 2 Department of Mathematics, Faculty of Physics, Mathematics and Optometry, University of Latvia, Jelgavas Street 3, LV-1004 Riga, Latvia; dmitry.gromov@lu.lv

**Keywords:** vision relaxation exercises, computer users, asthenopic complaints, vision examination, eye roll device

## Abstract

This study aims to explore the potential of a novel EYE ROLL device designed to facilitate guided vision relaxation exercises in an open space. A prospective study was performed on 89 participants who perform screenwork for at least four hours daily. All participants were randomly divided into three groups: a Control group with no exercising, a Manual group undertook manual vision relax ation exercises, and an Eyeroll group engaged in EYE ROLL device-assisted vision relaxation exercises. Each participant underwent three evaluations (an initial baseline assessment, a 4-week follow-up, and an 8-week follow-up) with four assessment tools: a comprehensive vision examination, an in-depth questionnaire, saccadic eye movement recordings, and objective accommodation measurements. There was a statistically significant decrease (35% and above) in complaint scores at the 4-week follow-up in both training groups. Although statistically insignificant, complaints continued to decrease after an 8-week period. No significant changes were observed in clinical or objective accommodative parameters. Some variation of visual functions was observed in all groups due to repeated measures. Vision relaxation exercises combined with proper vision ergonomics and working habits can reduce asthenopic complaints. The EYE ROLL device presents a promising tool for integrating these exercises into the working environment.

## 1. Introduction

With the rise in technological advancements, an increasing number of individuals report discomfort when using computers. This has led to an increase in asthenopic symptoms such as eye fatigue, eye pain, visual discomfort, dry eyes, watering eyes, and redness, collectively referred to as “Computer Vision Syndrome” (CVS). According to a review by Rosenfield [1], up to 90% of computer users may exhibit some CVS symptoms. To address eye fatigue and related issues, it is crucial to maintain proper visual ergonomics. This encompasses setting an optimal computer screen distance of 50–63 cm, ensuring appropriate screen contrast to reduce eye strain, having room lighting that is slightly brighter than the screen’s luminance, and taking regular breaks to rest the eyes, such as following the 20–20–20 rule [2]. Additionally, the use of appropriate vision correction is advised, with a preference for glasses over contact lenses [2]. Lastly, vision relaxation exercises, often referred to as “eye yoga”, can be performed during breaks to primarily alleviate asthenopic symptoms [3,4].

One of the primary obstacles and challenges in advocating for vision relaxation exercises for computer users is the inconsistency in empirical evidence regarding their efficacy for visual functions. For instance, Watten et al. [5] observed a notable decline in a range of oculomotor functions—including positive and negative accommodation, as well as positive and negative relative vergence (often termed fusional reserves)—following an 8 h computer workday. This suggests that prolonged computer use diminishes the force-generating capacity of the ciliary (responsible for eye accommodation) and extraocular muscles of the human eye. That can lead to issues like nearwork-induced transient myopia and fusion difficulties (manifesting as diplopia, eye strain, and eye fatigue), which vision relaxation exercises might alleviate. Conversely, Nyman et al. [6] found no significant changes in fusional reserve after a 5 h computer session. Moreover, they observed no notable differences in accommodative amplitude, convergence capacity (near point of convergence), and refraction when compared to a control group. Since no improvement of any vision complaints was observed, Nyman et al. [6] could not advocate for the necessity of vision relaxation exercises.

A limited number of studies have documented changes in clinical measurements following vision relaxation exercises. Gupta and Aparna [7] reported a statistically significant improvement in several parameters, including binocular accommodative facility, distance and near exophoria, both subjective and objective near points of convergence, vergence facility, and distance and near positive and negative fusion reserves, in participants after six weeks of eye yoga exercises. Thus, they argued that eye yoga exercises enhance vergence-related binocular vision functions by optimizing the efficiency of ocular muscles. Furthermore, they observed a reduction in asthenopic symptoms and a decrease in the prevalence and incidence of non-strabismic binocular vision anomalies. Desai et al. [8] detected small refractive error changes following a four-week vision relaxation regimen, though these changes were not statistically significant. In contrast, Abdel-Rahman Mohmed [9] observed a statistically significant reduction in refractive error among myopic patients following a six-week vision relaxation regimen.

Various methods exist for performing vision relaxation exercises. These include sourcing instructions online, viewing instructional videos, utilizing specialized computer software (e.g., Eye Relaxing and Focusing), employing computer reminders to schedule breaks (e.g., EyeDefender, Eye Relax, ProtectYourVision, Time Out, Eyeleo, Safe Eyes Linux), or leveraging smartphone applications that guide users through specific relaxation exercises (e.g., Eye exerises, Eye Buddy). Such software and applications typically present fixation stimuli or specially designed images on a screen as relaxation targets or provide written or illustrated step-by-step exercise instructions on how to relax the eyes. However, this methodology appears to conflict with the recommendation for computer users to shift their gaze away from screens for optimal relaxation. Completing the recommended exercises appropriately is another obstacle in avoiding overtaxing of the visual system. Consequently, it might be advantageous to employ off-screen instructions and undertake exercises by interacting with the surrounding environment. In light of this, the development of the EYE ROLL device (SIA EyeRoll, Kandava, Latvia) was motivated by society’s interest in utilizing different assistive devices and the lack of available instruments to aid in performing effective vision relaxation exercises. We explored the potential of a novel EYE ROLL device designed to facilitate guided vision relaxation exercises in an open space. The primary objective of this study was to assess the efficacy of the EYE ROLL device in alleviating vision strain among computer users, focusing on changes in asthenopic complaints and visual functions.

## 2. Materials and Methods

### 2.1. Participants

**Figure 1:**
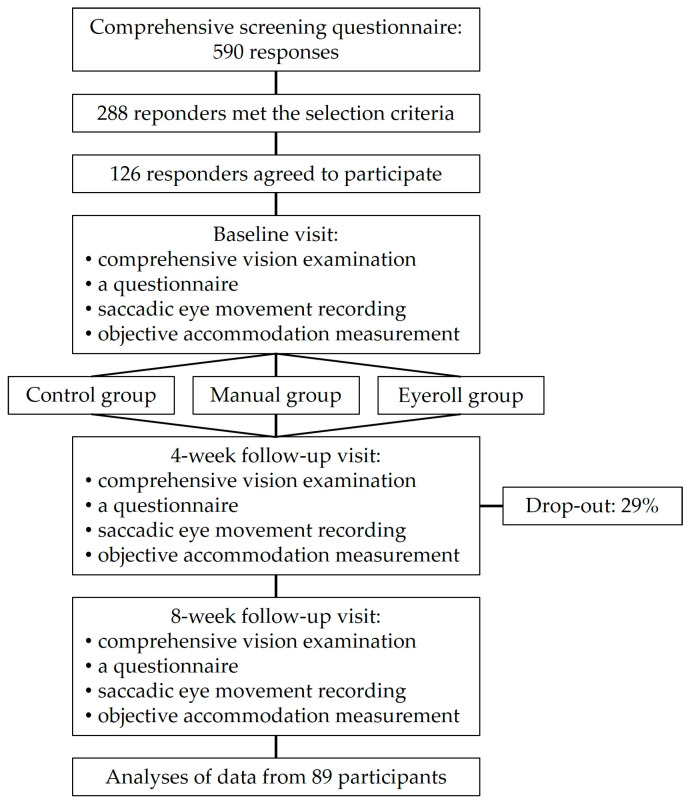
The diagram illustrates the study design and participant flow. For more details, see the text.

A total of 590 computer users provided responses to a comprehensive screening questionnaire capturing demographics, vision correction, and histories of ocular and general health. Of these, 288 respondents met the selection criteria: aged between 14 and 50 years, engaged in computer work for a minimum of four hours daily, a best corrected visual acuity (BCVA) of 0.8 (in decimal units) for both near and distant vision, and a non-corrected visual acuity of near 0.4 (in decimal units) or higher (to be able to perform objective accommodation and saccadic measurements). Individuals with conditions such as strabismus, nystagmus, or other ocular or systemic diseases were excluded. Finally, 126 respondents, with an average age of 31 ± 7 years (ranging from 17 to 50 years), participated in the study (see Figure 1).

Participants were randomly allocated into three groups: a control group that did not perform any vision relaxation exercises, a manual training group (Manual group) that undertook manual vision relaxation exercises, and an Eyeroll training group (Eyeroll group) that performed the EYE ROLL device-assisted vision relaxation exercises. Follow-ups were scheduled at 4-week and 8-week intervals. Thus, each participant was planned to have three visits: an initial baseline and two follow-up visits (a 4-week follow-up and an 8-week follow-up).

Finally, 89 participants (with 29% drop out) had at least one follow-up visit and were considered for statistical analysis (Control group—20 participants, Manual group—47 participants, Eyeroll group—22 participants). The size of the groups was not equal due to (1) limited number of EYE ROLL devices and (2) participants’ initial interest in performing vision relaxation exercises. Thus, the Control group was created from participants that either decided not to perform any exercises at the baseline visit or honestly admitted (during the 4-week follow-up) that no vision relaxation exercises were performed.

All study procedures were performed in accordance with the ethical standards of the institutional and/or national research committee. The Life and Medical Sciences Research Ethics Committee of the University of Latvia granted approval for the study, ensuring compliance with the 1964 Declaration of Helsinki and its subsequent amendments or equivalent ethical guidelines. Prior to their involvement, all participants provided written informed consent.

### 2.2. Vision Relaxation Exercises

**Figure 2:**
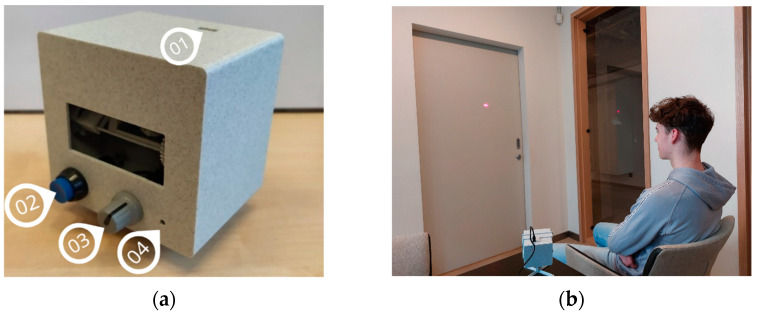
(**a**): The prototype of the EYE ROLL device used in the study for the Eyeroll group: (1) USB socket; (2) control button to pause the exercise; (3) handle to adjust projection screen amplitude; (4) element for velocity adjustment. (**b**): Example of position of a participant and a device during vision relaxation process.

Both the Manual group and Eyeroll group engaged in similar vision relaxation exercises, though they were performed using different methods (see Table 1): the Manual group had written instructions on how to perform vision relaxation exercises; the Eyeroll group had visually guided vision relaxation exercises using the novel EYE ROLL device (see Figure 2a), designed to facilitate a series of exercises analogous to manual vision relaxation techniques. The stimulus for vision exercises was a red laser beam that was projected by the device on a smooth surface. For effective utilization, the ambient lighting level should allow to the user to distinctly perceive the laser dot, with light-colored surfaces, such as grey or white, being ideal for projection to ensure contrast. The device, when placed on a level surface using its stand (Figure 2b), should be at least 2 m away from the projection surface, with both the user and device maintaining this distance. When projecting onto a wall, a seated position for users is recommended. The participants were instructed to keep their gaze on the laser dot whenever it appeared on the surface.

The laser dot’s movement amplitude in both horizontal and vertical directions can range from 10° to 50°. Although participants had the flexibility to adjust the amplitude based on the available projection area, the maximum amplitude was advised. A consistent stimuli speed of approximately 20°/s was set for all participants. To facilitate uninterrupted target tracking, the laser dot remained visible throughout the exercises, except for the far–near exercise. By pressing the control button (see Figure 2a), the participant could halt the training. When this button was pressed again, the training from the previously paused exercise was resumed.

The device employs a widened laser beam to produce a sizable point (~1.15° red laser dot), mitigating potential risks. The EYE ROLL features a Class 3R laser (650 nm, 5 mW), which is generally considered safe for the eyes. However, extended direct or reflected exposure can pose risks, while brief exposures or radiation dispersed from non-reflective surfaces, such as walls or doors, are considered harmless. Prior to initiating device utilization, participants were advised on safety precautions: (1) avoid direct gaze into the laser beam, and (2) ensure that the laser always points away from the user and other individuals and that the training surface is free from reflective objects and mirrors.

Participants in both training groups were instructed to undertake exercises for a minimum of 5–6 min, six times a day (total duration 30 min per day), on five days of the week. The participants were advised to take at least 5–10 min breaks every one to two hours to perform the set of exercises. Additionally, they received guidance on vision ergonomics, including aspects like working distance, sitting posture, and lighting. Participants from the Control group, while not assigned any vision relaxation exercises, were also informed about the importance of breaks during computer usage and vision ergonomics.

To follow the compliance of participants, all participants in all three groups were provided with a compliance table to capture their daily activities, including computer work hours, break frequencies and durations, screen-free time before sleep, and hours spent sleeping. Additionally, both the Manual group and Eyeroll group recorded the daily occurrences of their vision relaxation exercises.

### 2.3. Assessment Tools

To provide comprehensive evaluation of the effects of visual relaxation exercises, this study employed an integrated approach that included four distinct assessment tools: a comprehensive vision examination conducted by qualified optometrists, an in-depth questionnaire capturing computer usage behavior and associated complaints, saccadic eye movement recordings via the EyeLink 1000 Plus (SR Research, Ottawa, ON, Canada), and objective accommodation measurements using the PowerRef 3 (Plusoptix Inc., Nuremberg, Germany) device.

### 2.4. Vision Examination

Across all visits, participants underwent a comprehensive vision examination that included objective refraction (dry autorefractometry), assessment of non-corrected visual acuity using the Snellen decimal chart, subjective refraction assessment, and evaluations of binocular functions both at distance and near. The latter included the Worth four-dot suppression test, stereovision assessed by the Ostenberg and Titmus tests, heterophoria measurement with the Maddox rod test, and both positive and negative fusional reserves measured with a prism ruler. Ocular motility was assessed through the near point of convergence using the RAF ruler, with the NSUCO method used for saccadic and smooth pursuit evaluation. Accommodative function evaluation included positive and negative relative accommodation, binocular accommodative facility (±2.00 D flipper or Wick method for participants 30 years or older [10]), the dynamic retinoscopy MEM method, and monocular accommodative amplitude. A careful examination of both anterior and posterior eye structures was performed to rule out any anterior or posterior ocular disorders, with particular attention to the dry eye syndrome. Normal values of visual functions and accommodative and non-strabismic binocular disorders were based on the criteria outlined by Scheiman and Wick [10].

### 2.5. Questionnaire

Each participant filled out a questionnaire prior to every visit. Before the initial baseline visit, a uniform questionnaire was administered to all participants, gathering information about their vision correction usage, work habits, environment, associated asthenopic complaints, and any prior experience with vision training. Participants were asked to quantify the occurrence of symptoms such as eye fatigue, eye pain, dryness, visual discomfort, focusing challenges, eye redness, and watery eyes (response options: never, few times a month, several times a week, every day). Before the 4-week and 8-week follow-up visits, participants received group-specific questionnaires. For the Control group, the questions focused on the frequency of breaks taken, subjective complaints, and their willingness to try vision relaxation exercises. Participants in the Manual and Eyeroll groups were asked about the frequency of breaks, subjective complaints, the regularity of their vision training sessions, any challenges faced, any changes in their subjective perceptions post-training, and their willingness to continue the use of vision relaxation exercises post-study. In addition to the questions asked for the Manual group, the Eyeroll group also received questions regarding their experience with the EYE ROLL device, focusing on feedback related to its design, portability, and functionality.

### 2.6. Assessment of Saccadic Movements

We evaluated if vision relaxation exercises can affect saccadic eye movements because they are the most frequently used eye movements during computer work. The study employed the EyeLink 1000 Plus (SR Research, Ottawa, ON, Canada), a high-resolution (500 Hz) infrared video-based eye-tracking system. This device detects the pupil using the dark pupil algorithm and captures corneal reflections, as described by Hutton [11]. Visual stimuli were presented on a BenQ Model XL2430B (BenQ Corporation, Taipei, Taiwan) monitor with a resolution of 1920 × 1080 and screen dimensions of 533 mm × 300 mm. The monitor was positioned 93 cm from the participant, and the eye tracking camera was adjusted to be 60 cm from the participant’s eye level. To ensure consistent head positioning during data acquisition, participants used a forehead-chin rest supplied by SR Research (Ottawa, ON, Canada). Measurements were performed without any visual correction (glasses or contact lenses). The main reason for the absence of saccadic movement results in some participants was due to eye makeup, like mascara or eyeliner, which interfered with calibration and validation processes, subsequently affecting saccadic measurement accuracy.

The saccadic stimulus and its associated sequences were created using the Experiment Builder program (SR Research, Ottawa, ON, Canada). The saccadic stimulus consisted of a black dot (RGB 0; 0; 0) with a diameter of 1°, set against a grey background (RGB 166; 166; 166) with an average luminance of approximately 80 cd/m^2^. The display sequence was designed as follows: each slide presented a singular point, placed either horizontally (5° or 10°) or vertically (3° or 6°) from the center of the screen. The sequence initiated with a central cross displayed for two seconds, succeeded by the two-second stimulus presentation. Subsequently, the central cross slide reappeared. The stimuli were presented in three sequences, with each sequence containing all eight saccadic stimuli, arranged in a randomized order.

Prior to data collection, participants received verbal instructions to maintain a steady head position and focus on sequentially appearing dots and crosses without any head movements. The participants were instructed to limit blinking during the saccadic movements (blinking was allowed during the fixation phase). Participants were also given the option to adjust their chair for optimal comfort. The recordings were conducted in a well-illuminated room with an average illumination of 687 ± 3 lx (Illuminance Meter T-10WS, (Konica Minolta Sensing, Inc., Osaka, Japan)).

### 2.7. Assessment of Accommodative Response

Objective accommodation measurements were performed using the PowerRef 3 (Plusoptix Inc., Nuremberg, Germany), an eccentric photorefractometry device operating at a frequency of 50 Hz allowing measurement of refraction changes in steps of 0.01 D in the range of −7.00 D to +5.00 D at 20 ms intervals [12]. A printed white Maltese cross, with an angular size of 3° set against a black background, served as the far stimulus and was positioned 6.56 m away. The near stimulus, a printed 10 × 10 letter grid (black letters on a white backdrop, each letter corresponding to a visual acuity of 0.4 in decimal units), was set at a viewing distance of 0.4 m. The overall optical path for the photorefractometer camera was set to 1 m using a double mirror system as designed by Plusoptix Inc. (Nuremberg, Germany) [12]. A forehead and chin rest ensured a stable head position throughout the measurement procedure. Illumination during the measurement sessions was constant, with the room’s average illumination being 16.47 ± 0.45 lx (Illuminance Meter T-10WS, (Konica Minolta Sensing, Inc., Osaka, Japan)), although for some participants, lighting adjustments were made throughout the experiment. If a participant’s pupil size was too small, illumination was reduced until pupil size was sufficiently large to obtain data. If, after minimizing the illumination to the lowest level at which it was still possible to read the near stimulus, the pupil size remained too small for accurate measurements, the assessment was discontinued, and the participant did not complete the objective accommodation assessment.

Participants were instructed to alternate their fixation between distant and near stimuli. This cycle was repeated five times, with each fixation lasting 10 s, guided by an interval timer application that produced a sound signal every 10 s to guide the fixation change. Participants were asked to minimize blinking during the 10 s measurement and blink freely when changing focus between far and near stimuli.

### 2.8. Data Analysis

To simplify the evaluation of visual acuity, which also considers incorrectly named optotypes, visual acuity was converted from decimal units to LogMAR units. All participants (except one) had a subjective refraction ranging from −4.88 D to +3.75 D. One participant had a subjective refraction of −10.25 D for the right eye and −10.75 D for the left eye. This participant was included only in the analysis of questionnaire results.

From the questionnaire, information was collected about gender, age, vision correction (glasses, contact lenses, or both), working habits (including sleep duration, computer work hours, and frequency and duration of breaks during computer work), and asthenopic complaints. To analyze changes in specific complaints, the Likert Scale principles were applied. A complaint was classified as positive if the response was “never” or “rarely” and negative if the response was “often” or “very often”. Additionally, a complaint score was calculated for each participant using a 4-point scale: 0 = never experienced, 1 = rarely (a few times a month), 2 = often (several times a week), and 3 = very often (daily). The total complaint score was assessed by calculating the sum of seven items: eye fatigue, eye pain, dryness, vision discomfort, focusing problems, eye redness, and watery eyes. This score ranged from 0 to 21, with a higher score indicating a greater level of perceived complaints.

The results of saccadic movements for each participant during each visit (eye tracking session) were exported as Excel files from DataViewer (SR Research, Ottawa, ON, Canada). From the generated data file, we extracted the binocular amplitude (in degrees) and velocity (in degrees per second) of the first saccadic response. The results of three repetitions for each stimulus position were averaged. If the system recorded a blink during the saccadic movement, the results of this saccadic movement were excluded from the analyses.

The objective parameters of the accommodative response were obtained from the raw data file using a custom-made program implemented in MATLAB R2020a (MathWorks, Inc., Natick, MA, USA). Before data processing, all blinks were deleted from the raw dataset [13]. The raw data were divided in segments that corresponded to fixation at near and far objects. The results of the first and last seconds were deleted. The refractive state at the far and near fixation state was calculated only if 7–8 s of data records were available. Mean and standard deviation for each fixation state were calculated. The amplitude of the accommodative response (in dioptres, D) was calculated as the difference between the far and near refractive state [14]. The raw data were fitted to a Boltzmann sigmoidal function following the methodology described by Del Aguila-Carrasco et al. [14]. The peak velocity was calculated as the maximum or minimum of the sigmoid derivative, depending on whether it was accommodation (shifting fixation from far to near) or disaccommodation (shifting fixation from near to far), respectively [14]. The central 80% of the accommodative response was used to calculate the mean accommodative velocity [15]. Microfluctuations were expressed as a root mean square values (RMS), which is equal to the standard deviation of the refractive state [13,16]. To summarize, we calculated the accommodative response amplitude, standard deviation as representer of microfluctuations for near and distant fixation, as well as mean velocity and peak velocity for each accommodation and disaccommodation response of the right and left eye. The results of both eyes were averaged. The average value from all valid repetitions (up to five) was used for statistical analysis.

### 2.9. Statistical Analyses

Statistical analyses were conducted using the IBM SPSS software package version 20.0 and 22.0. The Shapiro–Wilk test, along with descriptive statistics and histograms, was used on all the data for normality testing. For data sets where 50% or more were normally distributed, we used the mean and standard deviation, as well as the parametric *t*-test and ANOVA (with Bonferroni post hoc test) [17]. If 50% or more of the data sets were not normally distributed, we applied median and interquartile range (IQR), as well as the Friedman test and Wilcoxon signed rank test (with Bonferroni correction) for related data [17]. Mixed-model ANOVA (with Mauchly’s test for sphericity and the Bonferroni post hoc test) was used to evaluate changes in parameters in relation to various affecting factors. McNemar’s exact test was used to evaluate changes in every complaint’s evaluation. Statistical significance was determined at a *p*-value of 0.05 or lower.

## 3. Results

The results of 89 participants were evaluated using the following assessment tools: (1) results of vision examination; (2) results of the questionnaires; (3) results of saccadic movements; and (4) results of accommodative response. There were several factors that limited the ability to collect all data for all participants; therefore, the number of participants in each assessment tool differed due to lack of data, such as not submitting or not fully completing questionnaires, missing compliance tables, or missing (or unanalyzable) saccadic or accommodative data. As a result, we analyzed data only for participants having the corresponding assessment tool data at the 4-week and 8-week follow-up visits and did not consider the need for a full set of assessment tools (see Table 2).

### 3.1. Results of Vision Examination

Looking at the whole set of visual function examination results, we noticed that there were participants with various refraction types and accommodative and/or non-strabismic binocular vision problems (see Table 3). Refractive error was considered based on subjective correction: (1) myopia without or with astigmatism; (2) emmetropia if spherical equivalent of subjective correction was in a range from −0.25 D to +0.25 D; (3) hyperopia without or with astigmatism. One participant with mixed astigmatism was classified in the myopia category. Diagnoses of accommodative or non-strabismic binocular vision disorder were based on criteria outlined by Scheiman and Wick [10]. Few participants had a combination of accommodative and non-strabismic binocular vision disorder; we classified them based on the primary problem.

More than 50% of most visual function evaluation data were not normally distributed (Shapiro–Wilk test, *p* < 0.05). Therefore, nonparametric tests were applied (see Table 4), except for phoria, accommodative amplitude, positive relative accommodation, and binocular accommodative facility that showed normal distribution.

All groups demonstrated some changes in non-corrected visual acuity after a 4-week follow-up: the Control group demonstrated small improvement in visual acuity for the left eye; the Manual group showed a decrease in visual acuity for the right eye but a small improvement in binocular visual acuity; the Eyeroll group showed improvement in visual acuity for both eyes. Results from the following visits showed no changes in visual acuity for the Control and Manual groups (Friedman test: *p* > 0.05). The Eyeroll group demonstrated no changes in visual acuity for the right eye and binocular visual acuity, but there was statistically significant improvement for the left eye by a few symbols. However, the Wilcoxon signed rank test (with Bonferroni correction for multiple comparisons) revealed no specific difference between visits.

None of the groups showed any changes in subjective correction at the 4-week follow-up. For the 8-week follow-up, only the Control group demonstrated small changes in spherical equivalent for the right eye; there was small shift toward more myopic correction and increased data distribution at the 4-week follow-up visit, but it cannot be considered as statistically significant if Bonferroni correction is applied for significance evaluation.

The mixed ANOVA test showed no changes in phoria at near and far for the 4-week follow-up. However, for the 8-week follow-up, there were statistically significant shifts in exophoria direction at the third visit both for distant (F(2, 112) = 40.417, *p* < 0.001) and near phoria (F(2, 112) = 5.429, *p* = 0.006), with no specific group difference. Pairwise comparison revealed that esophoric shift took place in both training groups for distant phoria (Manual group: *p* = 0.008, Eyeroll group: *p* < 0.001) and exophoric shift for near phoria (Manual group: *p* = 0.029; Eyeroll group: *p* = 0.022) but not in the Control group.

In the Control group, there were no changes in the near point of convergence and positive fusional reserves for the 4-week and 8-week follow-ups. Only negative fusional reserves both at far and near demonstrated a small improvement at the 4-week visit. However, no changes were observed for the 8-week follow-up visit. In the Manual and Eyeroll groups, no changes in the near point of convergence and fusional reserves were observed after the 4-week follow-up. In the Manual group, only positive fusional reserves showed improvement at distant and near fixation; the largest changes were at the 8-week follow-up visit for positive fusional reserve at distant fixation (Wilcoxon signed rank test with Bonferroni correction for multiple comparisons: Z = −2.694, *p* = 0.007) and both at the 4-week and 8-week follow-up visits at near fixation (Wilcoxon signed rank test with Bonferroni correction for multiple comparisons: 4-week: Z = −2.521, *p* = 0.012; 8-week: Z = −3.073, *p* = 0.002). In the Eyeroll group, only positive fusional reserves at near fixation showed small improvement. However, the Wilcoxon signed rank test with Bonferroni correction for multiple comparisons demonstrated no statistically significant difference between visits.

No changes were seen in negative and positive relative accommodation as well as binocular accommodative facility in any group after the 4-week and 8-week follow-ups. Accommodative amplitude showed significant improvement after the 4-week follow-up for the right eye (F(1, 75) = 4.452, *p* = 0.038) but not for the left eye; there were no differences between groups for both eyes (pairwise comparison: *p* > 0.05). For the 8-week follow-up, no difference was observed between visits and groups for both eyes. Thus, no accommodative parameter demonstrated any significant changes.

### 3.2. Questionnaires

Table 5 and Table 6 demonstrate the results of the complaint rates for all groups. In the Control group, there were no statistically significant changes in any of the complaint rates after the 4-week and the 8-week training period (McNemar Exact test, *p* > 0.05). In the Manual group, a statistically significant decrease (by applying the McNemar Exact test) was observed in several complaints already after the 4-week period: eye fatigue (*p*(2-tailed) = 0.001), dryness (*p*(2-tailed) = 0.002), and vision discomfort (*p*(2-tailed) = 0.006). The decrease in these complaints remained statistically significant also after the 8-week training period: eye fatigue (*p*(2-tailed) < 0.001), dryness (*p*(2-tailed) < 0.001), and vision discomfort (*p*(2-tailed) = 0.031). After the 4-week period, a decrease in the complaint rate for focusing problems was observed (*p*(1-tailed) = 0.035). However, in the group evaluated after the 8-week period, the complaint rate remained unchanged (*p*(1-tailed) = 0.063).

In the Eyeroll group, the largest decrease in complaints was observed in vision discomfort after 4 weeks (*p*(2-tailed) = 0.021). However, we were not able to observe this improvement after 8 weeks, where data from smaller group of participants was analyzed (*p*(2-tailed) = 0.22). After a 4-week training period, we observed a trend towards reduced eye fatigue complaints, without achieving statistical significance (*p*(1-tailed) = 0.063). A statistically significant reduction in eye fatigue complaints was observed after the 8-week training period (*p*(2-tailed) = 0.004).

The paired *t*-test was used to evaluate changes in the score of complaints in all groups and visits because the data were considered normally distributed. Changes in complaint scores in all groups (see Table 7) were analyzed by comparing the results from the baseline visit with either the 4-week follow-up visit or 8-week follow-up visit. For the Control group, no statistically significant change was observed at the 4-week visit or the 8-week visit. For the Manual and Eyeroll groups, we observed a statistically significant reduction in the complaint scores both at the 4-week visit and 8-week visit. For participants who had data from all three visits in the Manual group, the largest change in the complaints score was observed at the 4-week visit (ANOVA test for repeated measures: Mouchly’s Test of Sphericity, χ^2^(2) = 6.187; *p* = 0.045; Greenhouse–Geiser: F(1.63, 50) = 25.471, *p* < 0.001; Bonferroni post hoc; *p* < 0.001), with no significant differences in scores between the 4-week and 8-week visits. A similar tendency was observed in the Eyeroll group (ANOVA test for repeated measures: Mouchly’s Test of Sphericity, χ^2^(2) = 12.330; *p* = 0.002; Greenhouse–Geiser: F(1.26, 30) = 9.403, *p* = 0.004; Bonferroni host hoc; *p* < 0.001). No difference was observed between the Manual group and Eyeroll group in complaint score changes (mixed ANOVA, visit*group: F(1, 55) = 0.013, *p* > 0.05).

### 3.3. Saccades

More than 50% of the saccadic data demonstrated a normal distribution (see Table 8). Therefore, we used the mixed model ANOVA test (with the Bonferroni post hoc test) with group as a between-subject factor and visit and direction as within-subject factors.

At the 4-week follow-up visit, saccadic amplitude decreased at 10° to the right (Visit: F(1, 52) = 5.561, *p* = 0.022) and 6° down (Visit: F(1, 52) = 8.417, *p* = 0.005), with no significant difference between the groups. The other stimulus positions showed no significant changes. For those participants who had data both for the 4-week and 8-week follow-up visit, the minor difference that was previously observed at the 4-week follow-up visit did not persist at the 8-week follow-up visit.

Horizontal saccades showed asymmetry, with larger amplitudes to the right for both 10° (Direction: F(1, 52) = 22.398, *p* < 0.001) and 5° (Direction: F(1, 52) = 22.022, *p* < 0.001) stimuli, with no specific difference between groups. The horizontal asymmetry decreased; it remained at the 4-week follow-up visit and showed no statistically significant changes. A larger decrease was observed for 5° stimuli (Visit: F(1, 52) = 4.317, *p* < 0.043), with no difference between groups. The asymmetry remained after the 8-week period for the 10° (Direction: F(1, 40) = 32.554, *p* < 0.001) and 5° stimuli (Direction: F(1, 40) = 17.237, *p* < 0.001), with some fluctuations in between visits. However, the asymmetry fluctuation did not reach statistical significance between visits or groups. For vertical saccades, higher amplitudes were observed for downward movements at 6° and 3° stimuli (Direction: 6°: F(1, 52) = 4.179, *p* = 0.046; 3°: F(1, 52) = 5.397, *p* = 0.024), with no difference between groups. Only 6° stimuli (Visit: F(1, 52) = 4.241, *p* = 0.044) demonstrated slightly larger saccadic amplitudes at the baseline visit compared to the 4-week follow-up visit in all groups. The 8-week follow-up visit revealed no asymmetry in vertical saccadic amplitude for any of the groups.

For most stimuli, velocity analyses showed no significant differences at baseline and 4-week follow-up, except for upward velocities at 6° (Visit: F(1, 52) = 4.522, *p* = 0,038) and 3° (Visit: F(1, 52) = 4.452, *p* = 0.040), with variation between groups (Visit × Group: 6°: F(2, 52) = 3.886, *p* = 0.027; 3°: F(2, 52) = 3.263, *p* = 0,046). For both stimulus positions, only the Eyeroll group demonstrated a decrease in velocities at the 4-week follow-up visit (6°: *p* = 0.001; 3°: *p* = 0.002). Looking downward at the 6° stimulus (Visit × Group: F(1, 52) = 3.446, *p* = 0.039), only the Manual group had a larger saccadic velocity at the 4-week follow-up visit (*p* = 0.032). After 8 weeks, the velocity increased for 10° to the left (Visit × Group: F(4, 80) = 2.688, *p* = 0.037) and decreased for 6° up (Visit × Group: F(4, 80) = 2.974, *p* = 0.024), primarily in the Eyeroll group (4-week 10°: *p* = 0.047; 6°: *p* = 0.001; 8-week 10°: *p* = 0.005; 6°: *p* = 0.003).

Horizontal velocity asymmetries were observed at both the 4-week and 8-week follow-up visits, with larger velocity to the right for 10° (Direction 4 weeks: F(1, 52) = 41.751, *p* < 0.001; 8 weeks: F(1, 40) = 38.462, *p* < 0.001) and 5° stimuli (Direction 4-week: F(1, 52) = 17.993, *p* < 0.001; 8-week: F(1, 40) = 10.457, *p* = 0.002). Only for the horizontal 10° stimulus did the Manual and Eyeroll groups have a larger asymmetry (with smaller velocity to the left) on the baseline visit (Visit × Direction × Group: F(4, 80) = 2.801, *p* = 0.031), and it decreased (but remained) on the following visits. Only the 6° stimuli, which had a higher upward velocity, showed vertical velocity asymmetries (Direction 4-week: F(1, 52) = 9.407, *p* = 0.003; 8-week: F(1, 52) = 6.577, *p* = 0.014). After 4 weeks (Visit × Direction × Group: F(2, 52) = 3.318, *p* = 0.044), the Manual group decreased velocity in looking up but the asymmetry remained (baseline: *p* = 0.001; 4-week: *p* = 0.045), and the Eyeroll group decreased velocity in looking up and eliminated asymmetry (baseline: *p* = 0.003; 4-week: *p* > 0.05).

### 3.4. Accommodation

We analyzed accommodation response parameters only at the 4-week follow-up visit due to the small number of useful data (see Table 9). The Control group demonstrated higher accommodative response amplitude both at baseline and the 4-week follow-up visit compared to the Manual group (Bonferroni post hoc: *p* = 0.003) and Eyeroll group (Bonferroni post hoc: *p* = 0.004). The 4-week training period caused a decrease in accommodation response amplitude (mixed ANOVA test: F(1, 34) = 5.033, *p* = 0.032), with differences between the groups (mixed ANOVA test: F(2, 34) = 3.266, *p* = 0.05). The Bonferroni post hoc test revealed significant changes only in the Control group, where accommodation response amplitude was significantly smaller at the 4-week follow-up visit compared to the baseline visit (*p* = 0.007). For both the Manual and Eyeroll groups, no changes in accommodation response amplitude were observed after the 4-week training period. No changes in microfluctuations (both at near and far fixation), peak velocity, and mean velocity were observed across visits or between groups. When comparing accommodation to disaccommodation responses, disaccommodation showed lower peak and mean velocities at both the baseline and 4-week follow-up visits across all groups (mixed ANOVA test: peak velocity F(1, 25) = 21.976, *p* < 0.001; mean velocity F(1, 25) = 32.533, *p* < 0.001).

## 4. Discussion

Our results demonstrate that vision relaxation exercises impacted the complaints of computer users. For the Control group, we did not observe any changes after the 4-week or 8-week period, while we observed a statistically significant decrease (35% and above) in complaint scores after the 4-week training period for both training groups (the Manual and Eyeroll groups), with no differences between the groups. Although not statistically significant, complaints continued to decrease further after the 8-week period (42% and above if compared to the baseline visit). This suggests that the EYE ROLL vision training device can provide comparable alleviation of complaints like eye fatigue and vision discomfort as achieved with manual training. These results correspond to the findings of Kim [3], Dharani et al. [18], and Gupta and Aparna [4]. In the study by Gupta and Aparna [4], participants, who performed a set of vision relaxation exercises called “eye yoga” five times a week for 6 weeks for an average of 30 min, reported reduced feeling of eye fatigue compared to the Control group, which experienced increased eye fatigue. Similar to our results, the complaints of eye fatigue decreased in participants after eight weeks of vision training but remained unchanged in the Control group in the study [3], where participants performed eye yoga exercises twice a week for one hour. During the one-hour vision training, participants were in a Shavasana pose for 20 min. Other studies [19] also employed the Shavasana pose during eye yoga exercises, showing a reduction in asthenopic complaints after 4 weeks. As can be seen from previous studies, there is a slight difference in the periods that demonstrate efficiency in vision relaxing exercises. There are studies having a 4-week period [19], 6-week period [4], and 8-week period [3]. Most of our participants performed exercises for 4 weeks, with less than half continuing for an additional 4 weeks to reach the 8-week training period. The most evident reduction in complaint scores occurred within the initial 4-week training period, suggesting that 4 weeks are enough to assess the efficiency of vision relaxation exercises.

Key limitations of this study include participant compliance and the accuracy of exercise execution. As the participants performed the exercises outside an optometrist’s office, it is not possible to guarantee the reported information on the frequency, duration, and accuracy of training. To improve compliance in cases where home-based therapy is prescribed, the optometrist should be careful when explaining and demonstrating the exercises to the patient to ensure that they are properly understood. The patient’s compliance is likely to also be strongly affected by the optometrist’s arguments and belief that the exercises can help to improve the patient’s condition. Although we used compliance tables to follow the progress of our participants, 26% of participants did not submit the tables after finishing the 4-week training period.

Several additional factors could be related to the decrease in complaints. First, complaints and discomfort are greatly related to our sleeping habits. Insufficient sleep can cause dry eyes, redness, fatigue, and decrease in tear layer thickness [20]. People aged 18 to 60 require a minimum of 7 h of sleep daily to maintain optimal health [21]. In our study, it was observed that 53.7% of the participants had insufficient sleep duration (5–7 h). The hormone melatonin is responsible for the regulation of our sleep patterns. Its production is affected by exposure to blue light, which is a visible part of the spectrum, with wavelengths between 380 nm and 500 nm, commonly emitted by electronic devices. Increased exposure to blue light reduces melatonin production. A deficiency in melatonin can lead to insomnia and other sleep disorders, which can have significant repercussions on health and overall quality of life. Therefore, it is advisable to avoid the use of electronic devices before bedtime and to keep them away from the sleeping area [22].

Second, changes in the participant’s subjective complaints might also be related to the placebo effect. The placebo effect is a complex phenomenon, often based on a psychological aspect, caused by hope and trust. The participant’s confidence in the vision specialist and the treatment prescribed may lead to a complex cascade of biochemical reactions that may further affect the course of treatment and the perception of symptoms. Among the factors that lead to the formation of hopes and opinions, the most important is “verbal suggestion” [23]. Due to the placebo effect, it is difficult to evaluate the effectiveness of some treatments used by optometrists, such as correcting astigmatism or anisometropic spectacles in patients with persistent headaches, prismatic correction in patients with heterophoria, prism prescribing in migraine patients, and ocular motor training in patients with learning disabilities. The results of the above therapies may be more dependent on the placebo effect than on the physiological effect. A study [24] found that the placebo treatment program for convergence insufficiency was effective. Participants were divided into two groups: one group underwent specific vision exercises for convergence insufficiency, while the other engaged in placebo exercises that did not impact the accommodative and vergence system. The authors found no statistically significant difference between the groups after therapy. Thus, we cannot rule out the placebo effect in our study, given that participants acquired new knowledge and experience about working habits, ergonomics, and vision exercises. It is important to note that even though that the Control group also adopted these habits in our study, they did not report improvement in complaints. For example, our study demonstrated that implementation of vision relaxation exercises ensured that participants in the Manual and Eyeroll groups had more regular breaks during their working day. However, changing working habits alone cannot explain the decrease in complaints as multiple factor regression was applied. Thus, vision relaxation exercises complemented with regular breaks together can more effectively reduce complaints among intensive computer users. This underscores the potential benefits of vision relaxation exercises.

Regarding the user experience of the EYE ROLL device, 82% of participants found it easy to use. The remaining participants pointed out that the main reason for having problems performing training was related to time management—they struggled to find an appropriate time for vision relaxation during a busy workday. After the 4-week training period, 37% of participants reported improvements (decrease of complaints), with this figure rising to 63% after the 8-week period. Slightly over half of the participants preferred to use the EYE ROLL device at home. The main difficulty in using it in office was in finding a free surface for laser dot projection. Notably, 92% of participants expressed intentions to continue the vision relaxation exercises post-study, and 77% would recommend the EYE ROLL device to their colleagues and friends.

Vision relaxation exercises gained popularity at the beginning of the last century. In 1919, a book entitled “The Bates Method for Better Eyesight Without Glasses” [25] was published by ophthalmologist W. Bates. The book described a theory of vision based on the fact that the main accommodation tool is not the lens but the external muscles of the eye, which are responsible not only for eye movements but also for the extension and shortening of the anterior and posterior surfaces of the eye. Bates introduced specialized exercises, claiming that they could “cure” myopia, hypermetropia, astigmatism, presbyopia, and strabismus. However, modern discoveries prove that Bates’ understanding of the functioning of the visual system was misleading and that the exercises he proposed could not change the refractive error [26]. We used subjective refraction as an indicator of refractive error as it provides a more accurate representation of the refractive state than dry autorefractometry. Our findings indicate that vision relaxation exercises have no effect on subjective refraction and, thus, cannot cure any true refractive error. However, we still cannot exclude the impact of accommodation overload on the observed refractive changes (especially in myopia cases) claimed by other studies [26]. Therefore, studies analyzing the impact of vision training on ocular refraction must consider wet retinoscopy results.

On follow-up visits, all groups exhibited slight variations in visual acuity, especially after the 4-week period. We converted visual acuity into LogMAR units, with one optotype equal to 0.02 LogMAR units. The changes seen were only in one to three symbols, which is not clinically significant and likely attributed to variations in repeated measurements, as repeatability directly impacts the reliability of the results obtained. For good repeatability, the measurements must be taken under the same conditions [27]. For example, our previous study [28] demonstrated large variation in measurements of vergence and fusional reserve on various weekdays and even during the day. If accommodative amplitude measurement is repeated with a 24 h difference, the repeatability coefficient is ±1.24 at a viewing distance of 33 cm, compared to ±0.99 at a viewing distance of 40 cm [29]. Other variables, like the specific time of day when measurements were taken, could also affect the results. To enhance reliability and assess any visual acuity changes after vision relaxation exercises, it is advisable to conduct a longer study with repeated measurements at the same conditions (including time of day).

Some studies [7,8,9] present arguments that vision relaxation exercises can change visual functions. Our results showed that vision relaxation exercises both in the manual and EYE ROLL performance did not affect clinical assessments such as accommodative amplitude, positive and negative relative accommodation, accommodation facility, heterophoria, near point of convergence, and positive and negative fusional reserves. None of the groups demonstrated any changes in more detailed analyses of objectively assessed accommodation parameters such as accommodative amplitude, peak velocity, mean velocity, and microfluctuations. Thus, we found no evidence that vision relaxation exercises can improve eyesight and affect visual functions in a way necessary to cure some functional problems of vision such as accommodative and non-strabismic binocular disorders.

The only unexpected changes in accommodative response amplitude after the 4-week period were observed in the Control group. The small number of participants may conceal the primary explanation. The objective accommodation assessment equipment (PowerRef3) has limitations, particularly the potential for measurement errors when using spectacle correction. Therefore, we made measurements either without correction or with contact lenses (if the participant already used them). Consequently, some measurements were either not possible or unreliable, limiting the number of participants with usable objective accommodation assessment results. Another factor affecting accommodation responses is the refraction-accommodation effect, where hyperopic participants accommodate more than emmetropic ones without correction, while myopic participants accommodate less, with a decreasing response as myopia increases. The Control group had more hypermetropic participants (4/6), resulting in higher accommodation values. In contrast, myopic participants dominated in both training groups (Manual: 12 myopia, 3 hyperopia, 7 emmetropia; Eyeroll: 6 myopia, 1 hyperopia, 2 emmetropia), leading to lower accommodation responses. Age variation among groups was not significant, as no participants over 40, where physiological decline in accommodation is noticeable, were included. The careful selection of participants with the required visual acuities further ensured that age-related decline did not affect the measurements. Therefore, the small sample size and uneven distribution of refractive errors among groups primarily account for the differences in accommodation responses in the control group after four weeks. To get a better idea of how objective parameters change after vision relaxation exercises, future studies should include more people with the appropriate vision correction, especially contact lenses, and make sure that the groups have an equal number of refractive errors. Additionally, studies should evaluate accommodation both before and after exercises on the same day and track changes over a longer period.

The age of participants varied significantly across groups compared to other similar studies [3,4,7], where participants were students with an age variation of 18–24 years. However, we believe this did not impact on the results. The strict inclusion criteria of our study ensured participants had a best-corrected visual acuity (BCVA) of 0.8 for both near and distant vision, no ocular or systemic diseases, and appropriate vision correction if necessary. This eliminated complaints due to lack of subjective refraction or presbyopia. Studies [30,31] indicate visual functions decline post-50, but our sample had only three participants over 40, with only one aged 50. Most participants were under 40. Additionally, a Spearman’s rank-order correlation revealed a weak but significant negative correlation between age and baseline complaint scores (r_s_ = −0.256, *p* = 0.017), indicating fewer complaints with age. In addition, younger participants showed less interest in vision relaxation exercises, while those over 30 were more engaged. Thus, we are confident that age variation did not influence our findings regarding complaints or visual function.

There are no studies demonstrating the impact of vision relaxation exercises on objective eye movement parameters. Consequently, we evaluated some parameters of saccadic movements: quick conjugate movements of both eyes between fixation points. This is the primary eye movement used to explore the information on a screen. Our findings showed that the saccadic response amplitude varied slightly but with no difference between the groups. Thus, this variation was likely more related to repeated measures than to the use of vision relaxation exercises. However, when using the EYE ROLL device, vision relaxation exercises had an impact on the saccadic response’s velocity in both horizontal and vertical directions; velocity increased for the 10° to the left stimulus and decreased for the 6° up stimulus. On the baseline visit, saccades showed statistically significant asymmetry for both velocity and amplitude across all groups. The rightward saccade was larger and faster compared to the leftward saccade. Such saccadic response asymmetry was also described in previous studies [32,33]. Following the implementation of eye relaxation exercises, both training groups experienced a decrease in asymmetry in both the horizontal and vertical saccadic responses, specifically in terms of velocity. While we cannot establish a direct causal relationship between these changes and the decrease in complaints, and we cannot rule out that such changes are due to random fluctuations in the measurement, there can be some relation to the exercises used. The EYE ROLL device provides visually guided stimuli that can be compared to other saccadic training stimuli [34]. Saccades are typically trained by projecting the stimuli on a computer screen [35,36,37] or onto a wall [38]. Similar to the EYE ROLL device, Knox and Wolohan [38] employed a red laser dot (0.1°) that was projected at a distance of 1.5 m onto an almost white wall. The majority of saccadic training is carried out horizontally [35,36,37,39]. However, Bibi and Edelman [40] found that training saccades in one direction can enhance performance in other directions as well. The training can combine both smooth pursuit and saccadic movements [35]. The authors assumed that after a training session, the generation of saccades becomes more automatic and requires less effort. Therefore, further research is needed to explore the potential of the EYE ROLL device in enhancing eye movements beyond its role in vision relaxation.

## 5. Conclusions

Our study demonstrates that vision relaxation exercises combined with proper vision ergonomics and working habits can reduce asthenopic complaints. The EYE ROLL device presents a promising tool for integrating these exercises into the workday. Such exercises are particularly beneficial for screen users, especially those with screen time exceeding four hours daily and those experiencing symptoms of Computer Vision Syndrome, such as eye irritation, transient blurred vision, headaches, muscle fatigue, visual fatigue, etc. Based on our findings, it is important to note that vision relaxation exercises do not enhance visual functions like accommodation, visual acuity, or binocular functions, nor do they change the refraction errors. Our study demonstrates a clinically significant reduction in visual complaints already after a four-week training period. In addition, this study shows previously unpresented analyses of objective accommodative response and saccadic response in relation to vision relaxation exercises. We demonstrated the positive effect of visually guided exercises provided by the EYE ROLL device on saccadic asymmetry both in horizontal and vertical directions. To conclude, our results give new insight into understanding the impact of vision relaxation exercises on our visual system. Nevertheless, conducting further studies with a more equitable allocation of participants between the groups, examining the long-term impacts, tighter control of study conditions for more reliable measurements, and incorporating supplementary objective assessments of visual discomfort would enhance our comprehension of the impact of vision relaxation exercises on the visual system.

## Figures and Tables

**Table 1:** Description of vision relaxation exercises for the Manual group (Manual vision relaxation exercises) and the Eyeroll group (EYE ROLL guided vision relaxation exercises).

Type of Exercise	Manual Vision Relaxation Exercises	EYE ROLL Guided Vision Relaxation Exercises
Blinking	Blink gently several times for a duration of 50 s.	-
Horizontal movements	Slowly move the eyes from the left side to the right side and back. Repeat several times for 50 s.	Follow the horizontally moving laser point on the projection surface for 10 repetitions.
Rotational movements	Slowly move your eyes clockwise and counterclockwise (imagine that you draw a circle with your eyes). Repeat several times for 50 s.	Follow the rotational movement of the laser point on the projection surface, with five repetitions each for clockwise and counterclockwise movement.
Diagonal movements	Begin by directing your gaze to the upper left corner, then slowly move your sight to the lower right corner. Subsequently, shift the gaze to the upper right corner and then to the lower left corner. Repeat several times for 50 s.	Follow the movement of the laser point on the projection surface from the upper left corner to the lower right corner, then to the upper right corner and to the lower left corner for 10 repetitions.
Vertical movements	Slowly move your eyes straight up, then straight down, as if drawing a straight line with your gaze. Repeat several times for 50 s.	Follow the vertically moving laser point on the projection surface for 10 repetitions.
Far–near exercise	Fixate on a distant object (beyond 6 m) ensuring clear vision for 10 s, then shift focus to a near object (such as a pen tip or finger) approximately 40 cm away, maintaining clear vision for another 10 s. Alternate between far and near fixation 5 to 10 times.	Find and look at the laser dot for 5 s as it randomly appears on the projection surface. Once it disappears, shift your gaze to near (approximately 40 cm away) for another 5 s. Repeat this cycle 10 times.

**Table 2:** Number of participants analyzed for each assessment tool among the groups.

Type of Data Set	Control	Manual	Eyeroll
Gender (Female/Male)	15/5	43/4	12/10
Age (years) *	25 (11)	31 (12)	35 (12)
Number of participants’ results analyzed for different assessment tools (1/2/3) **			
Visual functions	15/7/12	45/33/34	20/19/21
Questionnaires	10/5/11	37/26/31	20/16/17
Compliance table	8/5/7	41/28/28	17/16/18
Saccades	11/7/8	28/21/23	16/15/19
Accommodation response	7/2/6	22/5/7	9/6/10

* Median and interquartile range (in brackets) are given for data that violated normal distribution of the data (Shapiro–Wilk test). ** Distribution of data in different analysis subtypes: 1—at 4-week follow-up visit (two following results), 2—at 4-week and 8-week follow-up visits (three following results), and 3—at 8-week follow-up visit (results of the first and third visit).

**Table 3:** Distribution of refractive error and accommodation or non-strabismic binocular vision disorders among the groups.

	Control N = 20	Manual N = 47	Eyeroll N = 22
Refractive disorder *			
Myopia *	8	28	13
Emmetropia	4	12	7
Hyperopia	8	7	2
Accommodation problems	3	9	1
Non-strabismic binocular vision disorders	2	11	8

* Myopia was considered without or with astigmatism. One participant with mixed astigmatism was classified in the myopia category. Emmetropia was considered if spherical equivalent of subjective correction was in a range from −0.25 D to +0.25 D. Hyperopia was considered without or with astigmatism.

**Table 4:** Visual function examination results at initial baseline, 4-week follow-up, and 8-week follow-up visits.

Visual Function		Control N = 15 (#7)	Manual N = 45 (#33)	Eyeroll N = 20 (#19)
Non-corrected VA (RE) (LogMAR)	Baseline	0.00 (0.34)	0.19 (0.82)	0.21 (0.68)
	4−week	0.02 (0.25)	0.22 (0.87) *	0.16 (0.58) *
	8−week #	0.09 (0.40)	0.22 (0.83)	0.15 (0.50)
Non-corrected VA (LE) (LogMAR)	Baseline	0.02 (0.46)	0.19 (0.86)	0.17 (0.47)
	4−week	−0.02 (0.38) *	0.26 (0.87)	0.14 (0.48) *
	8−week #	−0.08 (0.66)	0.26 (0.85)	0.10 (0.48) ¤
Non-corrected VA (BE) (LogMAR)	Baseline	−0.06 (0.28)	0.12 (0.86)	0.00 (0.52)
	4−week	−0.06 (0.23)	0.11 (0.86) *	0.00 (0.46)
	8−week #	−0.08 (0.33)	0.10 (0.88)	0.00 (0.48)
SE (RE) (D)	Range (Baseline)	−2.25 … +1.38	−4.88 … +3.38	−3.63 …+1.50
	Baseline	−0.25 (1.00)	−0.69 (1.91)	−0.44 (1.19)
	4−week	−0.25 (1.00)	−0.56 (1.72)	−0.38 (1.44)
	8−week #	0.13 (1.38) #	−0.50 (1.75)	−0.63 (1.25)
SE (LE) (D)	Range (Baseline)	−1.75 … +1.75	−4.38… +3.75	−3.75 … +1.50
	Baseline	−0.25 (1.01)	−0.69 (1.97)	−0.50 (1.13)
	4−week	−0.25 (1.13)	−0.69 (1.97)	−0.25 (1.38)
	8−week #	0.00 (2.00)	−0.25 (1.69)	−0.38 (1.25)
Phoria Far (Δ)	Baseline	0 ± 4	1 ± 3	0 ± 3
	4−week	1 ± 3	1 ± 3	0 ± 2
	8−week #	1 ± 4	2 ± 2	2 ± 3
Phoria Near (Δ)	Baseline	−4 ± 7	−2 ± 6	−4 ± 5
	4−week	−5 ± 5	−2 ± 6	−6 ± 5
	8−week #	−7 ± 5	−4 ± 6	−7 ± 6
NPC (cm)	Baseline	5.0 (2.5)	4.5 (1.5)	5.0 (1.3)
	4−week	5.0 (3.5)	4.5 (0.5)	4.5 (0.5)
	8−week #	5.0 (2.5)	4.5 (0.5)	5.0 (0.5)
NFR Far (Δ)	Baseline	8 (4)	8 (4)	8 (4)
	4−week	8 (2) *	6 (2)	8 (4)
	8−week #	6 (4)	8 (4)	8 (2)
NFR Near (Δ)	Baseline	14 (6)	10 (10)	10 (6)
	4−week	12 (8) *	12 (6)	12 (6)
	8−week #	12 (4)	10 (6)	12 (6)
PFR Far (Δ)	Baseline	25 (13)	16 (15)	17 (8)
	4−week	25 (21)	20 (11)	19 (13)
	8−week #	20 (2)	25 (10) ¤¤¤	20 (11)
PFR Near (Δ)	Baseline	25 (19)	25 (12)	20 (11)
	4−week	25 (17)	25 (10)	20 (16)
	8−week #	30 (15)	30 (14) ¤¤	30 (20) ¤
NRA (OD) (D)	Baseline	2.25 (0.75)	2.50 (0.50)	2.75 (1.00)
	4−week	2.25 (1.00)	2.50 (0.50)	2.25 (0.50)
	8−week #	2.50 (0.75)	2.75 (0.50)	2.50 (1.00)
PRA (OD) (D)	Baseline	3.07 ± 1.62	3.33 ± 1.56	2.46 ± 1.07
	4−week	2.93 ± 1.73	3.47 ± 1.40	2.46 ± 1.27
	8−week #	2.36 ± 0.78	3.15 ± 1.47	2.51 ± 1.29
BAF (cycles/min)	Baseline	8 ± 5	9 ± 6	9 ± 6
	4−week	10 ± 7	9 ± 5	8 ± 6
	8−week #	12 ± 4	10 ± 5	9 ± 7
AA (RE) (D)	Baseline	8 ± 3	9 ± 2	8 ± 2
	4−week	9 ± 4	9 ± 2	9 ± 3
	8−week #	8 ± 3	9 ± 2	8 ± 3
AA (LE) (D)	Baseline	10 ± 4	9 ± 3	8 ± 3
	4−week	10 ± 4	9 ± 3	9 ± 3
	8−week #	8 ± 3	9 ± 2	9 ± 3

# Data were calculated for the smaller number of participants that had data from all three visits (Control group—7; Manual group—21; Eyeroll group—15); they showed almost the same average data for the baseline and 4-week follow-up visits. Abbreviations: VA—visual acuity, RE—the right eye, LE—the left eye, BE—both eyes, D—diopters, Δ—prism diopters, NPC—near point of convergence, NFR—negative fusional reserves, PFR—positive fusional reserves, NRA—negative relative accommodation, PRA—positive relative accommodation, BAF—binocular accommodative facility, AA—accommodative amplitude. Phoria measurements: exophoria—negative sign, esophoria—positive sign. Wilcoxon signed rank test: * *p* < 0.05; Friedman test: ¤ *p* < 0.05, ¤¤ *p* < 0.01, ¤¤¤ *p* < 0.001.

**Table 5:** The rate of asthenopic complaints at baseline visit and after 4-week period.

Type of Complaint	Control				Manual				Eyeroll			
	Baseline		4-Week		Baseline		4-Week		Baseline		4-Week	
	P	N	P	N	P	N	P	N	P	N	P	N
Eye fatigue	4	6	4	6	14	23	28	9	8	12	12	8
Eye pain	10	0	8	2	36	1	36	1	16	4	19	1
Dryness	8	2	9	1	16	21	26	11	13	7	17	3
Vision discomfort	7	3	6	4	23	14	33	4	11	9	19	1
Focusing problems	8	2	7	3	26	11	32	5	15	5	19	1
Eye redness	8	2	8	2	31	6	30	7	16	4	17	3
Watery eye	9	1	10	0	34	3	35	2	18	2	18	2

Abbreviations: P—positive rate of complaints: observed never or rarely (a few times a month). N—negative rate of complaints: observed often (several times a week) or very often (every day).

**Table 6:** The rate of asthenopic complaints at baseline visit and after 8-week follow-up.

Type of Complaint	Control				Manual				Eyeroll			
	Baseline		8-Week		Baseline		8-Week		Baseline		8-Week	
	P	N	P	N	P	N	P	N	P	N	P	N
Eye fatigue	5	6	5	6	11	20	26	5	5	12	14	3
Eye pain	10	1	10	1	30	1	31	0	13	4	17	0
Dryness	7	4	9	2	15	16	27	4	11	6	15	2
Vision discomfort	11	0	9	2	22	9	28	3	9	8	13	4
Focusing problems	11	0	9	2	22	9	27	4	11	6	15	2
Eye redness	11	0	11	0	25	6	31	0	13	4	16	1
Watery eye	10	1	9	2	30	1	31	0	15	2	16	1

Abbreviations: P—positive rate of complaints: observed never or rarely (a few times a month). N—negative rate of complaints: observed often (several times a week) or very often (every day).

**Table 7:** A score of complaints (average ± standard deviation) in all groups separated by the analysissubtypes.

	Control	Manual	Eyeroll
Analyses 1	N = 10	N = 37	N = 20
Baseline	7.0 ± 3.2	7.4 ± 2.9	7.2 ± 4.1
4-week	6.8 ± 3.9	4.9 ± 3.4 ***	4.7 ± 3.2 ***
Analyses 2	N = 5	N = 26	N = 16
Baseline	5.4 ± 3.2	6.6 ± 2.8	7.6 ± 4.3
4-week	5.6 ± 4.2	3.9 ± 2.4	4.9 ± 3.3
8-week	4.0 ± 2.8	3.7 ± 2.3	4.4 ± 3.1
Analyses 3	N = 11	N = 31	N = 17
Baseline	5.7 ± 2.5	7.2 ± 3.3	7.5 ± 4.2
8-week	5.4 ± 3.3	4.0 ± 2.2 ***	4.2 ± 3.1 **

Analyses 1—all participants having data from the baseline visit and 4-week follow-up visit. Analyses 2—all participants having data from all three visits. Analyses 3—all participants having data only from the baseline visit and 8-week follow-up visit. Paired *t*-test: ** *p* < 0.01, *** *p* < 0.001.

**Table 8:** Parameters of saccadic response (mean ± standard deviation) at different stimulus positions.

		Control N = 11	Manual N = 28	Eyeroll N = 16	Average N = 55
		Response amplitude (°)			
10° to the right	Baseline	10.4 ± 0.7	9.9 ± 0.6	10.0 ± 0.4	10.0 ± 0.6
	4-week	10.1 ± 0.8	9.8 ± 0.7	9.2 ± 1.7	9.7 ± 1.1
	8-week *#*	10.6 ± 0.7	9.8 ± 0.5	9.7 ± 1.3	9.9 ± 0.9
10° to the left	Baseline	9.9 ± 0.7	9.3 ± 0.6	9.5 ± 0.5	9.5 ± 0.6
	4-week	9.9 ± 0.6	9.3 ± 0.7	9.3 ± 1.1	9.4 ± 0.8
	8-week *#*	9.7 ± 0.7	9.3 ± 0.5	9.3 ± 1.1	9.4 ± 0.8
5° to the right	Baseline	5.3 ± 0.5	5.1 ± 0.3	5.3 ± 0.3	5.2 ± 0.4
	4-week	5.3 ± 0.4	5.0 ± 0.7	4.9 ± 0.7	5.0 ± 0.6
	8-week *#*	5.4 ± 0.3	5.1 ± 0.4	5.0 ± 0.8	5.1 ± 0.5
5° to the left	Baseline	5.1 ± 0.4	4.7 ± 0.4	5.0 ± 0.4	4.9 ± 0.4
	4-week	4.9 ± 0.4	4.8 ± 0.5	4.8 ± 0.6	4.8 ± 0.5
	8-week *#*	5.2 ± 0.2	4.8 ± 0.4	4.9 ± 0.7	4.9 ± 0.5
6° up	Baseline	5.2 ± 0.4	5.3 ± 0.4	5.3 ± 0.7	5.3 ± 0.5
	4-week	5.3 ± 0.3	5.3 ± 0.7	5.2 ± 0.5	5.3 ± 0.5
	8-week *#*	5.4 ± 0.7	5.4 ± 0.7	5.0 ± 0.8	5.2 ± 0.7
6° down	Baseline	5.7 ± 0.8	5.5 ± 0.4	5.6 ± 0.6	5.6 ± 0.5
	4-week	5.4 ± 0.7	5.2 ± 0.7	5.3 ± 0.7	5.3 ± 0.7
	8-week *#*	5.4 ± 0.6	5.3 ± 0.4	5.3 ± 0.8	5.3 ± 0.6
3° up	Baseline	2.6 ± 0.6	2.7 ± 0.4	2.7 ± 0.4	2.7 ± 0.4
	4-week	2.6 ± 0.5	2.7 ± 0.4	2.7 ± 0.4	2.7 ± 0.4
	8-week *#*	2.8 ± 0.6	2.7 ± 0.4	2.8 ± 0.5	2.8 ± 0.5
3° down	Baseline	2.9 ± 0.5	2.8 ± 0.4	2.9 ± 0.6	2.8 ± 0.5
	4-week	2.9 ± 0.5	2.8 ± 0.6	2.8 ± 0.6	2.8 ± 0.6
	8-week *#*	2.7 ± 0.7	2.7 ± 0.3	2.8 ± 0.6	2.7 ± 0.5
		Mean velocity (°/s)			
10° to the right	Baseline	183 ± 11	170 ± 15	181 ± 8	175 ± 14
	4-week	181 ± 13	174 ± 11	180 ± 11	177 ± 12
	8-week *#*	184 ± 14	175 ± 14	177 ± 11	177 ± 13
10° to the left	Baseline	173 ± 11	157 ± 22	153 ± 21	159 ± 21
	4-week	174 ± 33	159 ± 20	167 ± 12	164 ± 22
	8-week *#*	173 ± 15	161 ± 14	169 ± 10	166 ± 14
5° to the right	Baseline	121 ± 11	118 ± 11	121 ± 8	119 ± 10
	4-week	124 ± 7	119 ± 9	119 ± 8	120 ± 9
	8-week *#*	125± 7	122 ± 8	119 ± 10	121 ± 9
5° to the left	Baseline	118 ± 7	107 ± 14	112 ± 21	110 ± 16
	4-week	120 ± 18	109 ± 14	115 ± 6	113 ± 13
	8-week *#*	122 ± 7	115 ± 9	116 ± 14	117 ± 11
6° up	Baseline	119 ± 12	131 ± 23	136 ± 27	130 ± 23
	4-week	121 ± 7	127 ± 24	119 ± 7	124 ± 18
	8-week *#*	123 ± 8	122 ± 13	118 ± 9	120 ± 11
6° down	Baseline	120 ± 17	116 ± 10	117 ± 11	117 ± 12
	4-week	116 ± 15	120 ± 10	116 ± 11	118 ± 12
	8-week *#*	121 ± 10	115 ± 11	116 ± 15	116 ± 12
3° up	Baseline	81 ± 11	89 ± 18	91 ± 21	88 ± 18
	4-week	82 ± 9	87 ± 16	79 ± 8	84 ± 13
	8-week *#*	84 ± 11	81 ± 6	82 ± 9	82 ± 8
3° down	Baseline	84 ± 12	78 ± 17	84 ± 14	81 ± 15
	4-week	85 ± 12	83 ± 7	80 ± 14	82 ± 11
	8-week *#*	84 ± 13	80 ± 6	84 ± 18	82 ± 12

# Data were calculated for the smaller number of participants that had data from all three visits (Control group—7; Manual group—21; Eyeroll group—15); they showed almost the same average data for the 4-week and 8-week follow-up visits.

**Table 9:** Parameters of accommodative response (mean ± standard deviation).

		Control	Manual	Eyeroll	Average
		N = 6	N = 22	N = 9	N = 37
Response amplitude (D)	Baseline	1.33 ± 0.51	0.64 ± 0.40	0.53 ± 0.28	0.72 ± 0.47
	4-week	1.07 ± 0.40	0.58 ± 0.36	0.57 ± 0.28	0.66 ± 0.38 *
		N = 6	N = 22	N = 9	N = 37
MF at near (D)	Baseline	0.11 ± 0.03	0.08 ± 0.03	0.07 ± 0.03	0.08 ± 0.04
	4-week	0.10 ± 0.04	0.08 ± 0.02	0.07 ± 0.02	0.08 ± 0.03
		N = 6	N = 22	N = 9	N = 37
MF at far (D)	Baseline	0.09 ± 0.05	0.05 ± 0.04	0.04 ± 0.04	0.06 ± 0.04
	4-week	0.07 ± 0.02	0.05 ± 0.03	0.05 ± 0.03	0.05 ± 0.03
		N = 7	N = 16	N = 6	N = 29
PV (Acc) (D/s)	Baseline	6.85 ± 3.29	4.17 ± 1.74	2.12 ± 1.08	4.39 ± 2.61
	4-week	5.89 ± 4.70	3.42 ± 1.70	1.99 ± 0.62	3.72 ± 2.87
		N = 7	N = 16	N = 6	N = 29
PV (DisAcc) (D/s)	Baseline	4.06 ± 2.39	1.93 ± 1.31	1.03 ± 0.85	2.26 ± 1.86
	4-week	3.13 ± 1.05	1.84 ± 1.27	1.11 ± 0.61	2.00 ± 1.29
		N = 7	N = 16	N = 6	N = 29
MV (Acc) (D/s)	Baseline	1.32 ± 0.45	1.30 ± 0.63	0.54 ± 0.40	1.15 ± 0.62
	4-week	0.97 ± 0.42	1.03 ± 0.57	0.64 ± 0.43	0.94 ± 0.52
		N = 7	N = 17	N = 6	N = 30
MV (DisAcc) (D/s)	Baseline	0.82 ± 0.43	0.65 ± 0.56	0.28 ± 0.14	0.61 ± 0.50
	4-week	0.66 ± 0.30	0.50 ± 0.28	0.36 ± 0.24	0.51 ± 0.29

Abbreviations: D—diopters, MF—microfluctuations, PV—peak velocity, MV—mean velocity, Acc—accommodation, DisAcc—disaccommodation. Results of mixed ANOVA: * *p* < 0.05.

## Data Availability

Data are available on request to the corresponding author.
